# Global Prevalence and Associated Clinical Markers of Thrombocytopenia in People Living with HIV: Evidence from Meta-Analysis

**DOI:** 10.3390/clinpract12060091

**Published:** 2022-10-31

**Authors:** Faisal K. Alkholifi, Sayed Aliul Hasan Abdi, Marwa Qadri

**Affiliations:** 1 Department of Pharmacology, College of Pharmacy, Prince Sattam Bin Abdulaziz University, Al-Kharj 11942, Saudi Arabia; 2 Faculty of Pharmacy, Al Baha University, Al Baha 1988, Saudi Arabia; 3 Department of Pharmacology and Toxicology, College of Pharmacy, Jazan University, Jazan 54943, Saudi Arabia

**Keywords:** HIV, CD < 200 cells/μL, thrombocytopenia

## Abstract

Thrombocytopenia is one of the complications in human immunodeficiency virus (HIV) patients. To improve the health outcomes of patients living with HIV, it is important to understand the prevalence and pattern of associated key clinical markers globally. This meta-analysis, therefore, aimed to estimate the pooled prevalence of and associated clinical marker of thrombocytopenia globally. Methodology: The meta-analysis was conducted as per Preferred Reporting Items for Systematic Reviews and Meta-Analyses (PRISMA) guidelines. All statistical analyses were conducted using Stata. Twelve full-text papers out of 454 were eligible for meta-analysis. Results: Among 6686 participants, overall pooled prevalence of thrombocytopenia was 10.90% (95% CI: 7.91, 13.88) I^2^ = 93.62%. In addition, thrombocytopenia was more prevalent by 25.11% (95% CI: 13.33, 36.88) in patients with CD + T < 200 cells/μL, and less prevalent in patients with CD + T < 200 cells/μL 10.10% (95% CI: 7.37, 12.83), respectively. Conclusions and recommendations: This meta-analysis established the prevalence of thrombocytopenia among patients living with HIV, and that it may be more prevalent in patients with CD + T < 200 cells/μL indicating the necessity of routine screening for various haematological markers and a careful treatment plan for HIV patients.

## 1. Introduction

Patients living with human immunodeficiency virus (HIV) may suffer from various haematological disorders, among which thrombocytopenia is very common [1,2]. However, serious bleeding complications and death have also been reported in HIV patients due to thrombocytopenia [3]. Furthermore, there is conflicting evidence of thrombocytopenia in HIV: for example, [4,5] have reported 5% and 59% cases of thrombocytopenia, respectively. In addition, HIV attaches itself to CD4^+^ T cell molecules and gradually decreases the number of circulating CD4^+^ T [6].

Thrombocytopenia in HIV patients has previously been related to the multifactorial, pathogenic mechanism [7]. Notwithstanding, the management of these conditions remains a clinical challenge for HIV specialists and haematologists [7], in spite of the fact that platelet transfusions may be required to manage complications. In addition, corticosteroids, intravenous immunoglobulins, and splenectomy are also used in patients depending upon the disease condition. However, these restorative modalities may be associated with several complications, such as HIV disease progression, infection transmission, and increased costs [8,9].

Numerous report of various haematological abnormalities in HIV patients have been published. However, there is a paucity of pooled prevalence data and associated haematological markers such as CD4^+^ T for thrombocytopenia in HIV patients, and pooled prevalence data may be helpful to develop health care policy and guidelines for preventive measures [10]. We hypothesize that cases of thrombocytopenia in HIV patients may be multifactorial, but different studies have different findings, for example, level of CD4^+^ T, age, and gender-specific vulnerability. Therefore, this study sought to investigate pooled prevalence by using evidence-based information available from different countries or regions reporting cases of thrombocytopenia in HIV.

## 2. Methods

### 2.1. Search Techniques and Criteria for Selection

This meta-analysis was conducted as per the guidelines of Preferred Reporting Items for Systematic Reviews and Meta-Analyses (PRISMA) [11,12]. All authors independently searched on databases PubMed, Embase, and Cochrane. A data extraction template was used to accumulate the information from each qualified study. We extracted the following data: study creator and nation, period of study, mean age, sample size, sample size for female and male, database utilized, reported number of HIV patients with thrombocytopenia, gender-wise segregation of male or female HIV patients affected by thrombocytopenia, and association of CD4^+^ T level for the development of thrombocytopenia. Each selected study’s data was independently abstracted by each author; if any disagreement was noted in respect of the data, then it was resolved by collective discussion. In, addition, the PRISMA checklist was used to present the findings of this systematic review and meta-analysis (refer to supplementary Table S1; Search strategy Table S2) [13]. For the study selection, we followed participants, intervention, comparators, outcomes, and study design (PICOS) criteria for the study selection (Table 1).

### 2.2. Statistical Analysis

A random effects analysis model was used to compute pooled prevalence rates of thrombocytopenia in HIV patients along with their 95% confidence intervals (CI). We used the recommended Tukey-Freeman arcsine transformed proportion and variance estimates for meta-analytic calculations of prevalence data, with the result presented as a forest plot [14]. Heterogeneity was assessed using the Higgins inconsistency index (I^2^), and if I^2^ > 50% it was considered substantial heterogeneity [15]. The odds of thrombocytopenia in HIV patients with CD4^+^ T levels at CD4+ T < 200 cells/μL compared to HIV patients with CD4+ T > 200 cells/μL were also assessed. A sensitivity analysis was used to investigate the effect of each study on the pooled prevalence by excluding each study in turn. Egger’s test and Begg’s test were used to assess publication bias [16]. All statistical analyses were computed using Stata (version 16.0; Stata Corporation, College Station, TX, USA).

### 2.3. Quality Assessment

The study’s quality was evaluated by using “The Joanna Briggs Institute Prevalence Critical Appraisal Tool”, which is a validated critical appraisal tool for systematic reviews addressing questions of prevalence [17].

## 3. Results

**Figure 1:**
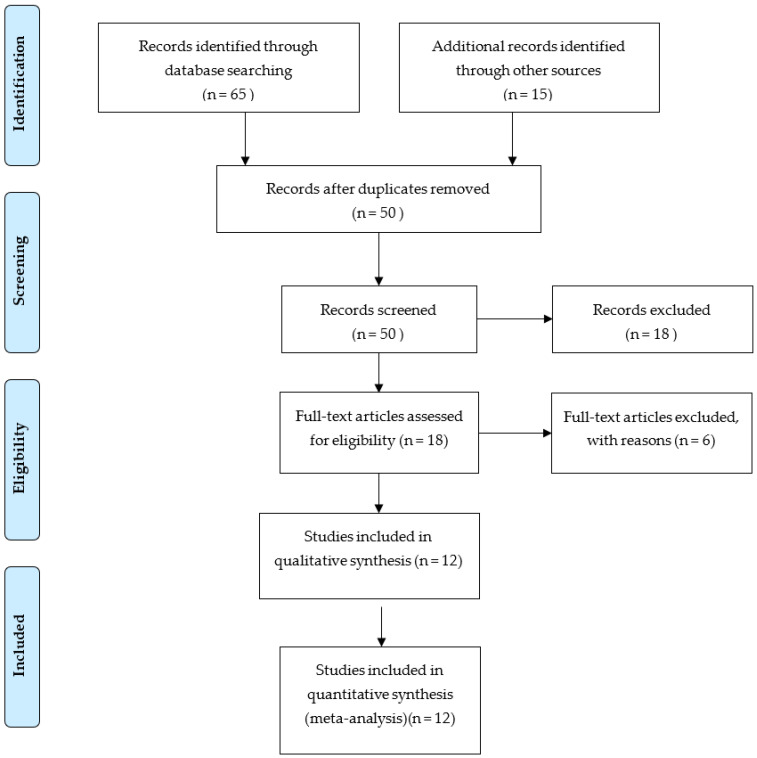
PRISMA diagram showing study screening process.

A total of 454 highly pertinent studies were found and examined. Of these, 45 studies were included by collaborative discussion with every author. The selected 45 studies were screened for their potential relevance to thrombocytopenia in HIV patients. On final screening, 12 cross-sectional studies were included for estimation of pooled prevalence in HIV patients suffering from thrombocytopenia. (Figure 1).

**Figure 2:**
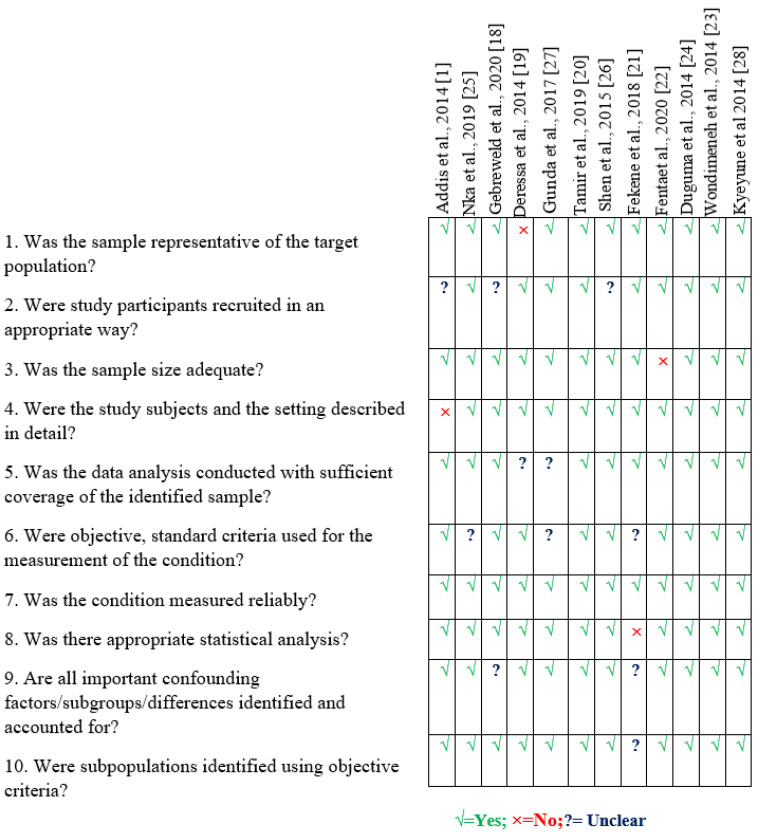
Risk of bias assessment using the Joanna Briggs Institute Prevalence Critical Appraisal Tool.

There were 6686 patients in total among 12 studies who were eligible. These studies were published from 2014 to 2021 and were conducted in Ethiopia (*n* = 8, 9.2% [1,18,19,20,21,22,23,24]), Cameroon (*n* = 1, 19% [25]), China (*n* = 1, 15.55%; [26]), Tanjaznia (*n* = 1, 14.44% [27]), and Uganda (*n* = 1, 8.25% [28]). Figure 2 shows additional quality scores for included studies.

### 3.1. Thrombocytopenia and Its Associated Clinical Markers in Adults Living with HIV

A total of 12 papers involving 6686 participants were included in this systemic review and meta-analysis, and all were used to compute the pooled prevalence of thrombocytopenia among adults living with HIV. The reported prevalence of thrombocytopenia in HIV patients varied from 4.03% to 19.03%. The lowest and highest prevalence of thrombocytopenia were 4.03% [22] and 19.03% [25], from Ethiopia and Cameroon, respectively.

### 3.2. Publication Bias and Sensitivity Analysis

Egger’s test and Begg’s test (*p* > 0.05) confirm that there is no publication bias. Due to the high degree of heterogeneity in the results, a sensitivity analysis was conducted, and each study was subsequently omitted or included one by one to assess the robustness of the pooled prevalence of thrombocytopenia in HIV patients. After the exclusion of any specific study, we did not see any significant change in the pooled prevalence of thrombocytopenia in HIV patients, revealing that thrombocytopenia is prevalent worldwide among HIV patients (Table 2).

## 4. Discussion

### 4.1. Main Findings

**Figure 3:**
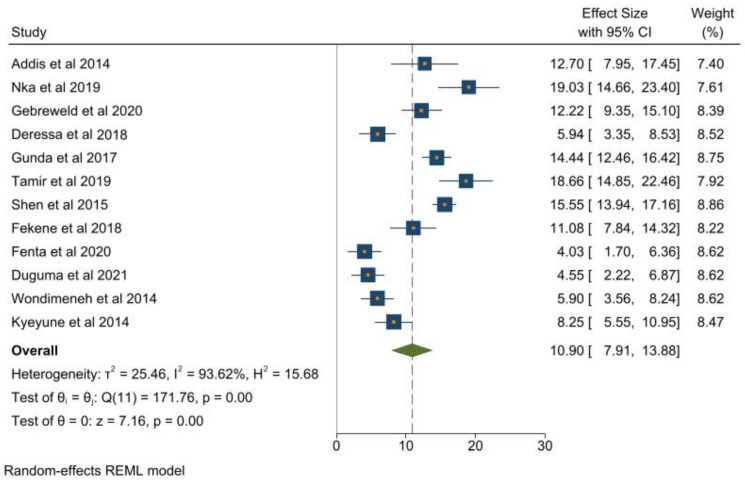
Pooled prevalence of thrombocytopenia among 6686 HIV patients from 12 studies [1,18,19,20,21,22,23,24,25,26,27,28].

In this meta-analysis, we reported pooled prevalence of thrombocytopenia in patients living with HIV (Figure 3). The pooled prevalence of thrombocytopenia was 10.90%, according to random effect analysis (95% CI: 7.91, 13.88).

### 4.2. Interpretation of the Findings

Our results suggest that thrombocytopenia in HIV patients is multifactorial and age, level of CD4^+^ T, and treatment modality may be considered as important cofactors. Out of 12 included studies, three studies [2,20,24] showed 4.03% to 5.90% prevalence of thrombocytopenia, which was lowest among the 12 included studies. However, [20,25,26] showed 19.03%, 18.66%, and 15.55%, respectively—the highest among included studies.

**Figure 4:**
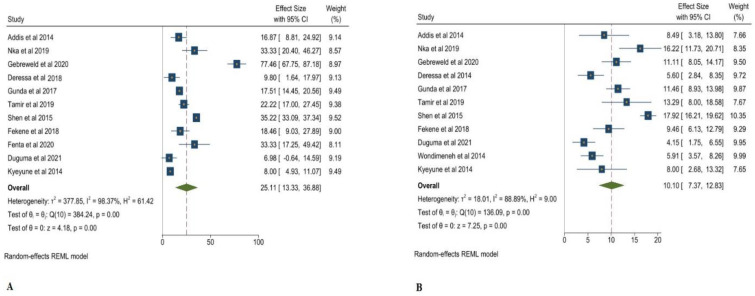
A Pooled prevalence of thrombocytopenia in CD + T < 200 cells/μL (**A**), CD + T > 200 cells/μL (**B**) [1,18,19,20,21,22,23,24,25,26,27,28].

In the study conducted by [22], mean age was 10.2 ± 3.2 years and CD4^+^ T > 350 cells/μL. Despite this, 73.7% were within 30–49 years of age, almost 51% of their CD4^+^ T levels were found to be >500 cells/μL, and only 13% of their CD4^+^ T levels were <200 cells/μL. In addition, Wondimeneh et al. [23] discovered that 71% of patients had CD + T < 350 cells/μL and more than 42% patients were under the age of 40 years. However, Nka et al. [25] found that almost 16% of patients had CD4^+^ T levels < 200 cells/μL and showed the highest percentage of thrombocytopenia, which was 19.03%. Tamir et al. [20] showed 60% patients had CD4^+^ T levels < 200 cells/μL and more than 41% of patients were under 39 years of age. In addition, as per the study of Shen et al. [26], almost 35% of patients had CD4^+^ levels < 200 cells/μL and 20% of patients were under 39 years of age. The overall prevalence of subjects with CD4^+^ T levels < 200 cells/μL was 25.11% (Figure 4A), and that of subject with CD4^+^ T levels > 200 cells/μL was 10.10% (Figure 4B).

**Figure 5:**
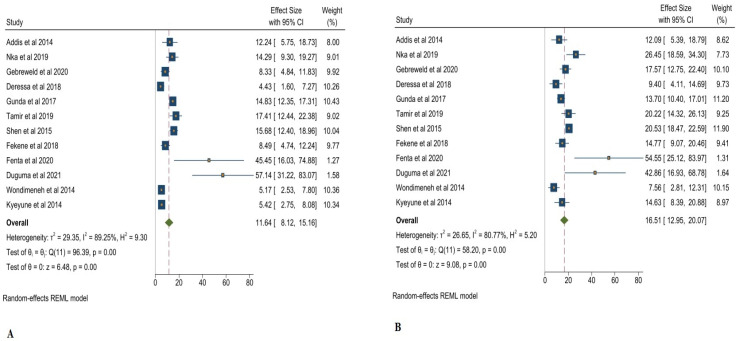
Pooled prevalence of thrombocytopenia in Female and Male. (**A**) Female, (**B**) Male [1,18,19,20,21,22,23,24,25,26,27,28].

**Figure 6:**
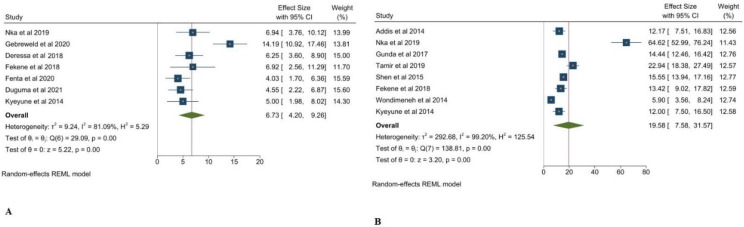
Pooled prevalence of thrombocytopenia in HAART subject (**A**), HAART naïve (**B**) [1,18,19,20,21,22,23,24,25,26,27,28].

It is already known that gender may play an important role in the progression of disease and decisions regarding epidemiological guidelines. In our meta-analysis it was observed that 11.64% were female and 16.51% were male, which reflects the fact that the preponderance of thrombocytopenia is higher in males (Figure 5A,B). Next, we have figured out the prevalence of thrombocytopenia with HAART therapy in comparison with HAART naïve. The preponderance of thrombocytopenia in HAART subjects was 6.73% in comparison with HAART naïve at 19.58% (Figure 6A,B). The decrease in the prevalence of thrombocytopenia with HAART treatment may be because of the platelet-restoring efficacy of HAART [29]. Furthermore, it has been found that an appropriate antiretroviral therapy is the most important step to fight thrombocytopenia in HIV patients [30]. Albeit, haematological changes with HAART regimen with special inference to zidovudine-containing HAART has been reported earlier by [31]. This meta-analysis extends the evidence of Hirsche 1988 [32] that zidovudine treatment may increase the platelet levels in HIV patient, but does not support the relevance of the study conducted by Talargia et al. [33] that HIV patients with low CD4^+^ T count and zidovudine in their HARRT treatment regimen may have high risk of thrombocytopenia, because our study concludes that CD + T < 200 cells/μL may be associated with thrombocytopenia, so it is difficult to say that a zidovudine-including HAART regimen is the only predisposing factor for thrombocytopenia in HIV patients. Indeed, in the study of Deressa et at. [19], a total 52% of patients were on a zidovudine HAART regimen, 49% on a Stavudine-based HARRT regimen, and 31.8% were on a Tenofovir-based HAART regimen. However, the highest number of patients were on a zidovudine HAART regimen, and only 6.3% got thrombocytopenia.

## 5. Implication

Routine assessment for thrombocytopenia along with evaluation of CD4^+^ T level in subjects living with HIV should be performed to optimize clinical management.

## 6. Conclusions

This meta-analysis based on real-world evidence suggests that a majority of HIV patients may have a vulnerability of thrombocytopenia, which may be because of multifactorial reasons.

## 7. Recommendations

The physician should critically evaluate the associated factors such as age, CD4^+^ T level, and treatment modality which may enhance the risk of thrombocytopenia in HIV patients.

## Figures and Tables

**Table 1:** PICOS criteria for study selection.

Parameters	Inclusion Criteria
Participants	HIV patients (aged 18 years or older)
Interventions	❖ Thrombocytopenia based on CD4^+^ T level. ❖ HAART and HAART naïve based categorization of thrombocytopenia. ❖ Gender based categorization of thrombocytopenia.
Comparators	❖ Thrombocytopenia in CD + T < 200 cells/μL vs. CD + T > 200 cells/μL. ❖ Thrombocytopenia in HAART vs. HAART naïve. ❖ Thrombocytopenia on the basis of gender (Male vs. Female).
Outcomes	Event of thrombocytopenia
Study designs	Cross sectional study

**Table 2:** Sensitivity analysis of the included studies to estimate the pooled thrombocytopenia in HIV patients.

Study Omitted	Estimated (95% CI)	Heterogeneity	
		I^2^ *p*-Value	
Addis et al. 2014 [1]	10.76 (7.54–13.98)	99.67	≤0.001
Nka et al. 2019 [25]	10.22 (7.32–13.12)	93.08	≤0.001
Gebreweld et al. 2020 [18]	10.22 (7.53–14.05)	94.26	≤0.001
Deressa et al. 2014 [19]	11.36 (8.23–14.49)	93.64	≤0.001
Gunda et al. 2017 [27]	10.56 (7.37–13.76)	93.52	≤0.001
Tamir et al. 2019 [20]	10.22 (7.31–13.13)	93.03	≤0.001
Shen et al. 2015 [26]	10.44 (7.31–13.57)	92.79	≤0.001
Fekene et al. 2018 [21]	10.89 (7.63–14.16)	94.38	≤0.001
Fenta et al. 2020 [22]	11.53 (8.56–14.51)	92.81	≤0.001
Duguma et al. 2021 [24]	11.49 (8.47–14.51)	93.03	≤0.001
Wondimeneh et al. 2014 [23]	11.37 (8.24–14.49)	93.50	≤0.001
Kyeyune et al. 2014 [28]	11.15 (7.92–14.39)	94.11	≤0.001
Combined	10.90 (7.91–13.88)	93.62	≤0.001

## Data Availability

Not applicable.

## Supplementary Materials

The following supporting information can be downloaded at: https://www.mdpi.com/article/10.3390/clinpract12060091/s1, Table S1: Search strategy for PubMed; Table S2: Preferred Reporting Items for Systematic Reviews and Meta-Analyses (PRISMA) checklist; Table S3: Characteristic of study included in the study.
