# Supplementary material for: Global Prevalence and Associated Clinical Markers of Thrombocytopenia in People Living with HIV: Evidence from Meta-Analysis
Source: Clin Pract. 2022 Oct 31;12(6):867–75. doi: 10.3390/clinpract12060091 (PMC9680325; doi:10.3390/clinpract12060091)
Supplement: Supplementary file 1 [file clinpract-12-00091-s001.zip › clinpract-1958481-supplementary.pdf]

Table S1. Search strategy for PubMed.

Searches	Search terms
#1	(((Human immunodeficiency virus, HAART Naïve, (HAART thrombocytopenia [Title/Abstract]) OR (CD4 hematological parameters[Title/Abstract])) OR hematological abnormalities, Zidovudine based HAART regimen, HIV patients[Title/Abstract])
#2	(((HAART, HAART Naïve, thrombocytopenia[MeSH Terms]) OR (hematological abnormalities, HIV,CD4 [MeSH Terms])) OR (CBC, Blood profile, Zidovudine [MeSH Terms])) OR (hematological abnormalities [MeSH Terms])
#3	#1 OR #2
#4	((((Human immunodeficiency virus [Title/Abstract]) OR (HIV[Title/Abstract])) *Cluster of differentiation 4, Male, Female, Zidovudine* thrombocytopenia OR (AIDS[Title/Abstract])) OR (HIV/AIDS [Title/Abstract])) OR ((HAART Naïve, [Title/Abstract])

Table S2. Preferred Reporting Items for Systematic Reviews and Meta-Analyses (PRISMA) checklist.

Section/topic	#	Checklist item	Reported on page #
**TITLE**			
Title	1	Identify the report as a systematic review, meta-analysis, or both.	1
**ABSTRACT**			
Structured summary	2	Provide a structured summary including, as applicable: background; objectives; data sources; study eligibility criteria, participants, and interventions; study appraisal and synthesis methods; results; limitations; conclusions and implications of key findings; systematic review registration number.	2
**INTRODUCTION**			
Rationale	3	Describe the rationale for the review in the context of what is already known.	3-5
Objectives	4	Provide an explicit statement of questions being addressed with reference to participants, interventions, comparisons, outcomes, and study design (PICOS).	5
**METHODS**			
Protocol and registration	5	Indicate if a review protocol exists, if and where it can be accessed (e.g., Web address), and, if available, provide registration information including registration number.	5
Eligibility criteria	6	Specify study characteristics (e.g., PICOS, length of follow-up) and report characteristics (e.g., years considered, language, publication status) used as criteria for eligibility, giving rationale.	5-6

Information sources	7	Describe all information sources (e.g., databases with dates of coverage, contact with study authors to identify additional studies) in the search and date last searched.	6
Search	8	Present full electronic search strategy for at least one database, including any limits used, such that it could be repeated.	6, Table S1
Study selection	9	State the process for selecting studies (i.e., screening, eligibility, included in systematic review, and, if applicable, included in the meta-analysis).	7
Data collection process	10	Describe method of data extraction from reports (e.g., piloted forms, independently, in duplicate) and any processes for obtaining and confirming data from investigators.	8
Data items	11	List and define all variables for which data were sought (e.g., PICOS, funding sources) and any assumptions and simplifications made.	8
Risk of bias in individual studies	12	Describe methods used for assessing risk of bias of individual studies (including specification of whether this was done at the study or outcome level), and how this information is to be used in any data synthesis.	8
Summary measures	13	State the principal summary measures (e.g., risk ratio, difference in means).	Not applicable
Synthesis of results	14	Describe the methods of handling data and combining results of studies, if done, including measures of consistency (e.g., I^2) for each meta-analysis.	8
Risk of bias across studies	15	Specify any assessment of risk of bias that may affect the cumulative evidence (e.g., publication bias, selective reporting within studies).	8
Additional analyses	16	Describe methods of additional analyses (e.g., sensitivity or subgroup analyses, meta-regression), if done, indicating which were pre-specified.	8
**RESULTS**			
Study selection	17	Give numbers of studies screened, assessed for eligibility, and included in the review, with reasons for exclusions at each stage, ideally with a flow diagram.	9, Figure 1
Study characteristics	18	For each study, present characteristics for which data were extracted (e.g., study size, PICOS, follow-up period) and provide the citations.	9, Table 1
Risk of bias within studies	19	Present data on risk of bias of each study and, if available, any outcome level assessment.	7, Table S3
Results of individual studies	20	For all outcomes considered (benefits or harms), present, for each study: (a) simple summary data for each intervention group; (b) effect estimates and confidence intervals, ideally with a forest plot.	Not applicable
Synthesis of results	21	Present results of each meta-analysis done, including confidence intervals and measures of consistency.	10

Risk of bias across studies	22	Present results of any assessment of risk of bias across studies.	11
Additional analysis	23	Give results of additional analyses, if done (e.g., sensitivity or subgroup analyses, meta-regression).	11-13
**DISCUSSION**			
Summary of evidence	24	Summarize the main findings, including the strength of evidence for each main outcome; consider their relevance to key groups (e.g., healthcare providers, users, and policy makers).	13-14s
Limitations	25	Discuss limitations at study and outcome level (e.g., risk of bias), and at review level (e.g., incomplete retrieval of identified research, reporting bias).	13
Conclusions	26	Provide a general interpretation of the results in the context of other evidence, and implications for future research.	13
**FUNDING**			
Funding	27	Describe sources of funding for the systematic review and other support (e.g., supply of data); role of funders for the systematic review.	Not applicable

Background color: xxxxx.

Table S3. Characteristic of study included in the study.

Study	Location	Study design	Outcome Thrombocytopenia in participants Event / Total	Gender Male Event/ Total	Gender Female Event/ Total
Addis et al 2014	Ethiopia	Cross sectional study	24/ 189	11/91	12/98
Nka et al 2019	Cameroon	Cross sectional study	59/ 310	32/121	27/189
Gebreweld et al 2020	Ethiopia	Cross sectional study	61/ 499	42/239	20/240
Deressa et al 2014	Ethiopia	Cross sectional study	19/ 320	11/117	9/203
Gunda et al 2017	Tanzania	Cross sectional study	174/ 1205	57/416	117/789
Tamir et al 2019	Ethiopia	Cross sectional study	75/ 402	36/178	39/224
Shen et al 2015	China	Cross sectional study	303/ 1948	303/1476	74/472
Fekene et al 2018	Ethiopia	Cross sectional study	40/ 361	22/149	18/212
Fenta et al 2020	Ethiopia	Cross sectional study	11/ 273	6/11	5/11
Duguma et al 2021	Ethiopia	Cross sectional study	14/ 308	6/14	8/14

Wondimeneh et al 2014	Ethiopia	Cross sectional study	23/ 390	9/119	14/271
Kyeyune et al 2014	Uganda	Cross sectional study	33/400	18/123	15/277
